# Immune-related adverse events in patients with pre-existing autoimmune rheumatologic disease on immune checkpoint inhibitor therapy

**DOI:** 10.1186/s41927-022-00297-5

**Published:** 2022-11-08

**Authors:** Amanda Lusa, Carolina Alvarez, Shruti Saxena Beem, Todd A. Schwartz, Rumey Ishizawar

**Affiliations:** 1 Division of Rheumatology, Allergy, and Immunology, University of North Carolina, Chapel Hill, NC USA; 2 Thurston Arthritis Research Center, University of North Carolina, Chapel Hill, NC USA; 3 Department of Biostatistics, Gillings School of Global Public Health and Thurston Arthritis Research Center, University of North Carolina, Chapel Hill, NC USA

**Keywords:** Immune-related adverse event, Immune checkpoint inhibitor, Rheumatologic disease, Anti-rheumatic therapy, Rheumatoid arthritis, Disease flare

## Abstract

**Introduction:**

Immune checkpoint inhibitors (ICIs) enhance the immune system’s ability to target and destroy cancer cells, but this non-specific immune overactivation can result in immune-related adverse events (irAEs). Patients with underlying autoimmune diseases were excluded from the original ICI clinical trials because of the theoretical risk of irAEs. This study aimed to evaluate the risk of irAEs in patients with pre-existing rheumatologic diseases on ICIs, impact of anti-rheumatic therapy on irAEs, and malignancy outcomes.

**Methods:**

We performed a retrospective chart review of patients with a pre-existing rheumatologic diagnosis receiving ICIs at the University of North Carolina from 2014 to 2019. Risk differences (RD) and asymptotic 95% confidence intervals (95% CIs) using a continuity correction along with odds ratios (OR) and exact 95% CIs were produced between pre-specified risk factors and flares of the underlying rheumatologic disease and/or irAEs. Kaplan–Meier survival estimates for time to unfavorable cancer response between subsets of patients were compared using the log-rank test.

**Results:**

We identified 45 patients receiving an ICI with an underlying rheumatologic diagnosis, including 22 with rheumatoid arthritis (RA). Overall, 13 patients (29%) had a flare of their autoimmune disease, 20 patients (44%) had a new-onset irAE, and 27 (60%) had either a flare or new-onset irAE. Patients with RA had higher risk of flares compared to those with other rheumatologic disorders (45% vs 13%, RD 32%, 95% CI 2.0–56.8); all RA flares were ≤ grade 2 and treated in the outpatient setting. Concurrent treatment of the rheumatologic disease at the start of ICI therapy was not associated with a reduced risk of flare (OR 0.86, 95% CI 0.19–3.76) or new onset irAE (OR 3.21, 95% CI 0.83–13.6) compared to those not on anti-rheumatic medications. Anti-rheumatic therapy did not impact time to unfavorable malignancy outcome (*p* = 0.52).

**Conclusion:**

The majority of our study cohort experienced a flare or new onset irAE with ICI treatment. Treatment with anti-rheumatic therapy did not prevent disease flares or new onset irAEs, but did not negatively impact malignancy outcomes. Research is needed to determine safe anti-rheumatic therapy options to prevent flares and irAEs that do not interfere with malignancy outcomes.

## Background

Immune related adverse events (irAEs) are unintended, although mechanistically predictable, consequences of immune checkpoint inhibitor (ICI) therapy [1]. The first approved ICI therapy was ipilimumab, a cytotoxic T-lymphocyte associated protein 4 (CTLA-4) antagonist, in 2011 for melanoma. Since then, this field has rapidly expanded with six additional ICIs targeting PD-1/PD-L1 pathways approved for treating a wide variety of malignancies both as first line and second line therapies [2]. Due to the immune modulation effects of ICIs and risk for irAEs, patients with underlying autoimmune diseases were excluded from the initial studies of ICI therapy. Presentations of irAEs can appear similar to rheumatologic disorders such as inflammatory arthritis [3], polymyalgia rheumatica, sicca symptoms [4], and myositis [5], suggesting that ICIs may increase the risk of disease flares in individuals with pre-existing rheumatologic disease. The prevalence of rheumatoid arthritis (RA) in the general population worldwide is about 0.5% [6]. Per the National Institute of Health Autoimmune Diseases Coordinating Committee Report from October 2000, the collective prevalence of all autoimmune conditions affects at least 5% of the United States population. Patients with rheumatologic autoimmune conditions also have a higher risk for malignancies compared to general population due to inherent altered immune systems as well as risks posed from immunosuppressive therapy to treat [7]. As ICI therapy can prolong survival in the setting of otherwise fatal malignancies, it is crucial to determine the true risks for irAEs and cancer outcomes in patients with underlying autoimmune disease who are indicated to receive ICI.

To date there have been several published case series and cohort studies looking at the incidence of irAEs and disease flares in patients with pre-existing autoimmune diseases, including those with rheumatologic conditions [8–15]. Several groups have shown flares in a majority of patients with RA [8, 11, 16, 17]. However, data regarding effects of immunosuppression on irAE and flare risk, as well as cancer treatment outcomes, is minimal and based on cohorts that include non-rheumatologic patients as well [9, 11, 14].

This study aimed to evaluate the risk of flares and irAEs in patients with underlying autoimmune rheumatologic disease treated with ICIs at the University of North Carolina (UNC) and to investigate the impact of anti-rheumatic therapy on the incidence of immune events and malignancy outcomes.

## Methods

### Patient selection

This study was conducted through retrospective chart review of patients with autoimmune rheumatologic diseases treated with ICIs at UNC from 2014 to 2019. This study was reviewed and approved by the Institutional Review Board of the University of North Carolina at Chapel Hill prior to start of data collection under IRB 17-1841. Due to the retrospective design and observational nature of the study, the requirement for informed consent was waived by the ethics committee of the University of North Carolina at Chapel Hill. Patients treated with ICIs at UNC were initially identified through i2B2 (Informatics for Integrating Biology and the Bedside) and the Carolina Data Warehouse for Health (CDW-H). The i2b2 is the flagship tool developed by the i2b2 Center, an NIH funded National Center for Biomedical Computing based at Partners HealthCare System, and CDW-H is a central data repository containing clinical, research, and administrative data sourced from the UNC Health Care System, with the ability to query most data elements as far back as mid-2004. The i2b2 linked with the CDW-H to query deidentified aggregate data to identify our cohort of patients on ICIs. Due to a transition in electronic medical records systems being used at UNC in 2014, clinical data from the legacy system was not included in this analysis despite FDA approvals for ICIs being available since 2011. Among 2842 patients treated with ICIs at UNC through December 31st, 2019, those with underlying rheumatologic diseases were identified using EMERSE (Electronic Medical Record Search Engine). EMERSE allows users to search free text (unstructured) clinical notes from the electronic health record [18]. In the EMERSE search we used these specific terms: “*rheumatoid arthritis”, “psoriatic arthritis”, “ankylosing spondylitis”, “polymyalgia rheumatica”, “sarcoidosis”, “lupus”, “sjogren’s”, “connective tissue disease”, “scleroderma”, “vasculitis”, “angiitis”, and “myositis”.* Chart review was performed in the electronic medical record. In total 45 patients were identified that carried a chart diagnosis listed above and had either been on or discussed treatment with a physician that supported their autoimmune diagnosis. There were an additional 34 patients with a chart diagnosis for rheumatic disease not supported by available records or a treatment plan who were excluded due to concern for misclassification.

In addition to rheumatologic diagnosis, we also extracted data on demographics (sex, age at malignancy diagnosis), ICI drug including anti-cytotoxic T-lymphocyte associated protein 4 (anti-CTLA4) therapy (ipilimumab), programmed cell death protein 1 (PD-1) inhibitors (pembrolizumab, nivolumab), or programmed death ligand 1 (PD-L1) inhibitors (atezolizumab, durvalumab), co-morbidities of interest (other autoimmune disease, prior malignancy, hypothyroidism, cardiovascular disease), anti-rheumatic therapy at the time ICI was started (hydroxychloroquine, disease modifying anti-rheumatic drugs (DMARDS), biologics, JAK inhibitors, corticosteroids, combination therapies), and autoimmune disease control before ICI therapy was started as documented by the oncologist. At the time of this study, the most recently approved ICIs - avelumab (2017) and cemiplimab (2018) - were not available to query in the i2b2 database and were excluded from analysis. Eastern cooperative oncology group (ECOG) score was also recorded but only available for 30 patients and was therefore not used in analysis because of the limited data set.

### Outcomes

Study outcomes of interest were occurrences of rheumatologic disease flares and new onset irAEs, and cancer outcomes as assessed by chart review.

Flares were defined by clinical documentation of an exacerbation of a patient’s underlying rheumatologic disease, and irAEs were defined by clinical documentation of a new-onset inflammatory responses separate from the patient’s underlying disease. All irAEs were categorized by common terminology criteria for adverse events (CTCAE) grade, either by the oncology care team or de novo by the study team if oncology notes did not specify. The specific irAEs were further classified by organ system. The time from the first dose of ICI to the onset of the flare or irAE was calculated. Information on treatment for the flare or irAE was collected, including the use of systemic corticosteroids, as was information on whether the ICI was held.

Cancer outcomes were ascertained though chart review of oncology notes and were noted as complete remission, partial remission, stable disease, mixed response, progression, or death as documented by the oncology team using RECIST criteria. During analysis, cancer outcomes were categorized as favorable (partial remission or stable disease) or unfavorable (mixed response, progression, or death). Subjects with a favorable cancer response (i.e., partial remission or no progression) were censored on the date of their last documented cancer response assessment after the start of ICI therapy. Subjects with an unfavorable cancer response (i.e., mixed, progression on therapy, progression off therapy, or death) were defined as having the event of interest, with the number of days determined from start of ICI therapy.

### Statistics

Descriptive statistics were used to summarize study patients and relevant variables. Counts and percentages were produced for categorical variables, while means and standard deviations (SD) were computed for continuous variables.

Odds ratios (OR) and exact 95% confidence intervals (95% CI) were produced between pre-specified risk factors and flares or irAEs. In addition, risk differences (RD), characterized as differences in percentages, and their corresponding asymptotic 95% CI using a continuity correction, were produced for certain risk factors (age, sex, rheumatologic disease type, anti-rheumatic treatment at the time of ICI therapy, and rheumatologic disease control) to aid in interpretation.

Kaplan–Meier estimates for time to unfavorable cancer response were produced and separately stratified for (1) incidence of a flare or irAE versus those without; (2) occurrence of a flare of irAE that required oral or intravenous steroids versus those without an event requiring systemic steroids; (3) ICI being held due to a flare or irAE versus continuing ICIs; (4) treatment for the rheumatologic disease during ICI treatment versus no rheumatologic treatment; (5) rheumatoid arthritis versus other rheumatologic disease. Kaplan–Meier estimates were produced by stratum and the log-rank test was used to assess for differences in time to unfavorable cancer response between levels of the strata described above.. All analyses were performed with SAS version 9.4 (SAS institute Inc., Cary, NC). Statistical significance was determined at an alpha level of 0.05.

## Results

We identified 45 patients with pre-existing autoimmune rheumatologic disease on ICI therapy, including 22 with rheumatoid arthritis, 5 with systemic lupus erythematous, 4 with polymyalgia rheumatica, 4 with psoriatic arthritis, and 10 with other conditions (Table 1). The mean (± SD) age was 68 (± 11.2) years. The majority of patients were white (*n* = 34, 76%) and female (*n* = 31, 69%). The most common indication for ICI was non-small cell lung cancer (NSCLC, *n* = 22, 49%). Most patients were treated with PD-1 inhibitors (*n* = 37, 82%).

**Table 1:** Baseline patient characteristics

Patient characteristic	n*	%*
Age, mean ± SD	67.9	± 11.2
Gender, female	31	68.9
*Race*		
White	34	75.5
Black or African American	8	17.8
Other^1^	3	6.7
*Rheumatologic disease*		
Rheumatoid arthritis	22	48.9
Lupus	5	11.1
Polymyalgia rheumatica	4	8.9
Psoriatic arthritis	4	8.9
Other^2^	10	22.2
*Malignancy type*		
Non-small cell lung cancer	22	48.9
Renal cell carcinoma	5	11.1
Melanoma	4	8.9
Other^3^	14	31
*Comorbidities*		
Other autoimmune disease^4^	4	8.9
Prior malignancy	10	22.2
Hypothyroidism	16	35.6
Cardiovascular disease	13	28.9
*Immune checkpoint inhibitor*		
Pembrolizumab	21	46.7
Nivolumab	16	35.6
Atezolizumab	5	11.1
Durvalumab	1	2.2
Ipilimumab	1	2.2
Nivolumab + pembrolizumab	1	2.2
*Anti-rheumatic therapy at initiation of ICI*	22	48.9
Hydroxychloroquine	11	24.4
DMARD^5^	4	8.9
JAK inhibitor^6^	1	2.2
Oral corticosteroid	3	4.4
Combination^7^	3	8.9
*Autoimmune disease controlled before ICI therapy*		
Yes	40	88.9
No	5	11.1
*Treatment line*		
1	19	42.2
2	17	37.8
3 or more	9	20.0

*ICI*, immune checkpoint inhibitor; *DMARD*, disease modifying anti-rheumatic drug; *JAK*, Janus kinase

*Unless otherwise specified

^1^Other races include Asian (*n* = 1) and unknown (*n* = 2)

^2^Other rheumatologic disease include ankylosing spondylitis (*n* = 2), dermatomyositis (*n* = 1), limited scleroderma (*n* = 2), sarcoidosis (*n* = 2), Sjogren's syndrome (*n* = 2), and undifferentiated connective tissue disease (*n* = 1)

^3^Other malignancy types include bladder cancer (*n* = 1), breast cancer (*n* = 3), endometrial carcinoma (*n* = 2), merkel cell carcinoma (*n* = 1), small cell lung cancer (*n* = 3), squamous cell carcinoma of the skin (*n* = 1), squamous cell of the tongue (*n* = 1), poorly differentiated lung cancer (*n* = 2)

^4^Other autoimmune diseases include autoimmune hepatitis (*n* = 1), Grave’s disease (*n* = 2), and psoriasis (*n* = 1)

^5^DMARDS include methotrexate (*n* = 2), sulfasalazine (*n* = 1), leflunomide (*n* = 1)

^6^JAK inhibitor includes tofacitinib (*n* = 1)

^7^Combinations include methotrexate and etanercept, hydroxychloroquine and prednisone, and tacrolimus and prednisone (for heart transplant)

The incidence of flare of the underlying rheumatologic disease was 29% (*n* = 13) and the incidence of new onset irAEs was 44% (*n* = 20), with a total of 60% (*n* = 27) of the patients experiencing either a flare or irAE (Table 2). Of the new onset irAEs, gastrointestinal events were most common (*n* = 7, 16%), followed by pulmonary events (*n* = 5, 11%). There were 3 new-onset rheumatologic irAE events in patients without those features as a part of their pre-existing disease. These included arthralgias in a patient with cutaneous discoid lupus, extensor tenosynovitis in a patient with polymyalgia rheumatica, and bone pain in a patient with Sjogren’s syndrome. Nine patients had multiple events, which could include a flare of their underlying rheumatic condition and/or new onset irAE events.

**Table 2:** Immune event outcomes

Outcome	n*	%*
Flare^1^	13	28.9
irAE^2^	20	44.4
Gastrointestinal	7	15.5
Pulmonary	5	11.1
Dermatologic	4	8.9
Endocrine	3	6.7
Rheumatologic	3	6.7
Other	2	4.4
Any event (flare or irAE)	27	60.0
Multiple events	9	20.0
Severe events (grade 3 +)	8	17.8
Death (grade 5 event)	1	2.2
Flare/irAE requiring systemic steroids	21	46.7
Unfavorable cancer response (M/PG/D)	37	82.2
Median time to unfavorable cancer response, median (95% CI) days	139	(98, 193)

*irAE*, immune related adverse event; *M*, mixed response; *PG*, progressive disease; *D*, death

*Unless otherwise specified

^1^Flare of the underlying rheumatologic disease as defined by clinical documentation of an exacerbation of a patient’s underlying rheumatologic disease

^2^New-onset immune related adverse event distinct from pre-existing disease

In terms of severity, 8 patients experienced a grade 3 or higher new onset irAE based on CTCAE criteria. One of the patients passed away from fulminant pneumonitis. All flares of the underlying disease were grade 2 or lower. The median (interquartile range) time to flare was 28 days (21–63), and to an irAE was 32 days (20–56). The majority of patients were treated with systemic oral or intravenous steroids for their flare or irAE (*n* = 21), while the minority did not require systemic steroids (*n* = 6) (Table 3). Regarding other immunosuppressive treatments for flares or irAEs, one patient with a grade 2 rheumatoid arthritis flare resumed hydroxychloroquine in addition to starting an oral corticosteroid, and one patient with grade 3 colitis required infliximab followed by vedolizumab in addition to corticosteroids. With the exception of the patient who passed from grade 5 fulminant pneumonitis, all patients with grade 3 or higher events saw a resolution in flare and/or irAE in response to holding ICI therapy and systemic steroids.

**Table 3:** Flare and irAE event management among those with a flare or irAE

	*n*	%
*Required systemic steroids*		
Yes	21	77.8
No	6	22.2
*ICI therapy held*		
Yes	16	59
No	11	41

We assessed whether ICI therapy was held at time of a flare of irAE. In total 16 of the patients who had an immune event required their ICI to be held (Table 3). ICI was held at the time of an immune event primarily for patients with grade 2 or higher events, though it was also held temporarily in one patient with grade 1 thyroiditis who required radiation, and permanently in another patient with grade 1 pneumonitis.

**Fig. 1:**
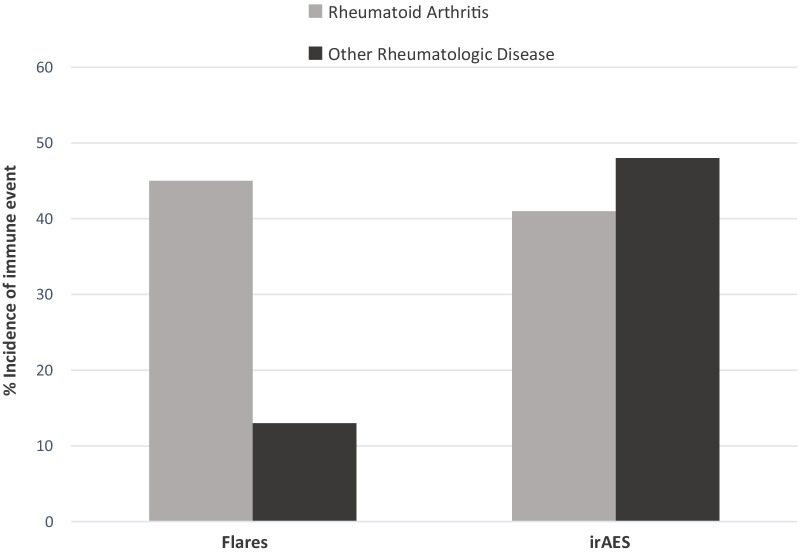
Incidence of flares and new onset irAEs on ICI therapy. A comparison of flares or irAEs triggered by ICI therapy among patients with rheumatoid arthritis versus other rheumatologic diseases

Associations between pre-specified risk factors and flares or new onset irAEs are shown in Table 4. The incidence of flares was higher in the RA population compared to those with other rheumatologic diseases (45% vs 13%, [OR 5.34, 95% CI 1.09–36.3], [RD 32%, 95% CI 2.0–56.8%], (Fig. 1)). However, the incidence of new onset irAEs was not higher in the RA group (41% vs 48%, [OR 0.76 95% CI 0.20–2.86], [RD 7%, 95% CI -23–36%]). Age and sex were not associated with the risk of flares or irAEs.

**Table 4:** Bivariate analysis of risk factors for flares and irAEs

	No flares *n* (%)	Flares *n* (%)	OR (exact 95% CI)	No irAEs *n* (%)	irAEs *n* (%)	OR (exact 95% CI)
*Rheumatologic disease*						
Other (*n* = 23)	20 (87)	3 (13)	Reference	12 (52)	11 (48)	Reference
RA (n = 22)	12 (55)	10 (45)	5.34 (1.09–36.3)	13 (59)	9 (41)	0.76 (0.20–2.86)
*Age*						
< 65 (*n* = 17)	12 (71)	5 (29)	Reference	12 (71)	5 (29)	Reference
≥ 65 (*n* = 28)	20 (71)	8 (29)	0.96 (0.21–4.65)	13 (46)	15 (54)	2.71 (0.66–12.6)
*Gender*						
Female (*n* = 31)	21 (68)	10 (32)	Reference	16 (52	15 (48)	Reference
Male (*n* = 14)	11 (79)	3 (21)	0.58 (0.08–2.93)	9 (64)	5 (36)	0.60 (0.13–2.56)
*Rheumatologic treatment*						
No (*n* = 23)	16 (70)	7 (30)	Reference	16 (70)	7 (30)	Reference
Yes (*n* = 22)	16 (73)	6 (27)	0.86 (0.19–3.76)	9 (41)	13 (59)	3.21 (0.83–13.6)
*Autoimune disease control before ICI therapy*						
No (*n* = 5)	4 (80)	1 (20)	Reference	1 (20)	4 (80)	Reference
Yes (*n* = 40)	28 (70)	12 (30)	1.70 (0.15–91.4)	24 (60)	16 (40)	0.17 (0.00–1.96)

Out of the 45 patients with pre-existing rheumatologic disease, 22 of these patients were on established anti-rheumatic treatment at the time ICI therapy was initiated. The most common anti-rheumatic treatment was hydroxychloroquine (*n* = 11, 24%) followed by DMARDs (*n* = 4, 9%), detailed in Table 1. There were no statistically significant effects seen in the odds of flares (OR 0.86, 95% CI 0.19–3.76) or new onset irAEs (OR 3.21, 95% CI 0.83–13.6) between those on versus off anti-rheumatic therapy (Table 4). Additionally, there were no associations between autoimmune disease control status before ICI therapy and risk of flares or irAEs (Table 4).

In Table 5, the frequencies of flares and irAEs, including severities and management, are further delineated by underlying rheumatic disease type. For each rheumatic disease type affected by flare or irAE, the majority had ICI therapy held or required systemic steroids. The majority of adverse events were of Grade 1 or 2, rather than of higher severity i.e., Grade 3 or higher (Tables 2 and 5). There was only 1 death clearly linked to an immune related adverse event (Table 2).

**Table 5:** Frequencies of features by rheumatic disease type (N = 45)

	RA (*n* = 22)	Lupus (*n* = 5)	PMR (*n* = 4)	PsA (*n* = 4)	AS (*n* = 2)	Limited SSc (*n* = 2)	Sarcoidosis (*n* = 2)	SS (*n* = 2)	DM (*n* = 1)	UCTD (*n* = 1)
Flare (*n* = 13)	10	0	0	1	1	0	0	1	0	0
irAE (*n* = 20)	9	3	2	1	2	0	1	1	0	1
AEs (Grade 1–2) (n = 19)	11	3	1	0	2	0	0	2	0	0
SAEs (Grade 3 +) (*n* = 8)	4	0	1	1	0	0	1	0	0	1
ICI therapy held (*n* = 16)	6	3	1	1	2	0	1	1	0	1
Required systemic steroids (*n* = 21)	12	1	2	1	2	0	1	1	0	1

*RA*, Rheumatoid arthritis; *PMR*, Polymyalgia Rheumatica; *PsA*, Psoriatic Arthritis; *AS*, Ankylosing Spondylitis; *SSc*, Scleroderma; *SS*, Sjogren's Syndrome; *DM*, dermatomyositis; *UCTD*, undifferentiated connective tissue disease; *irAE*, immune related adverse event; *AE*, adverse event; *SAE*, serious adverse event; *ICI*, immune checkpoint inhibitor

**Fig. 2:**
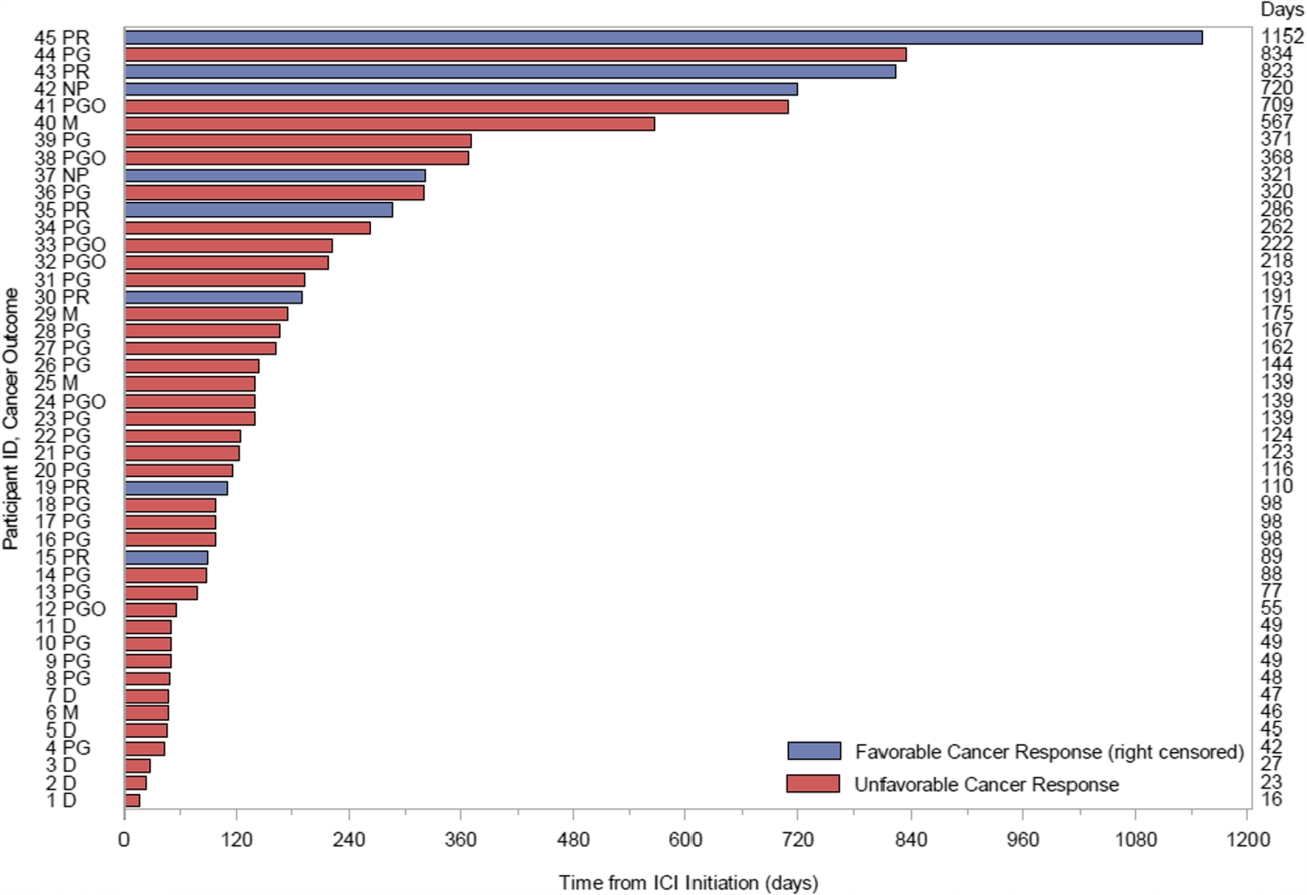
Cancer response to ICI therapy. Time to cancer outcomes from start of ICI therapy and onset of immune events (flare of underlying rheumatologic disease and/or irAE). Blue indicates favorable cancer outcomes defined as partial remission or stable disease and was censored on the date of their last documented cancer response assessment after the start of ICI therapy. Red indicates unfavorable cancer outcomes defined as mixed response, progression, or death and time to event of interest is reported in the number of days determined from start of ICI therapy. Cancer outcome abbreviations: CR = complete remission, PR = partial remission, NP = no progression, M = mixed response, PG = progressed on treatment, PGO = progressed off treatment, D = death

**Fig. 3:**
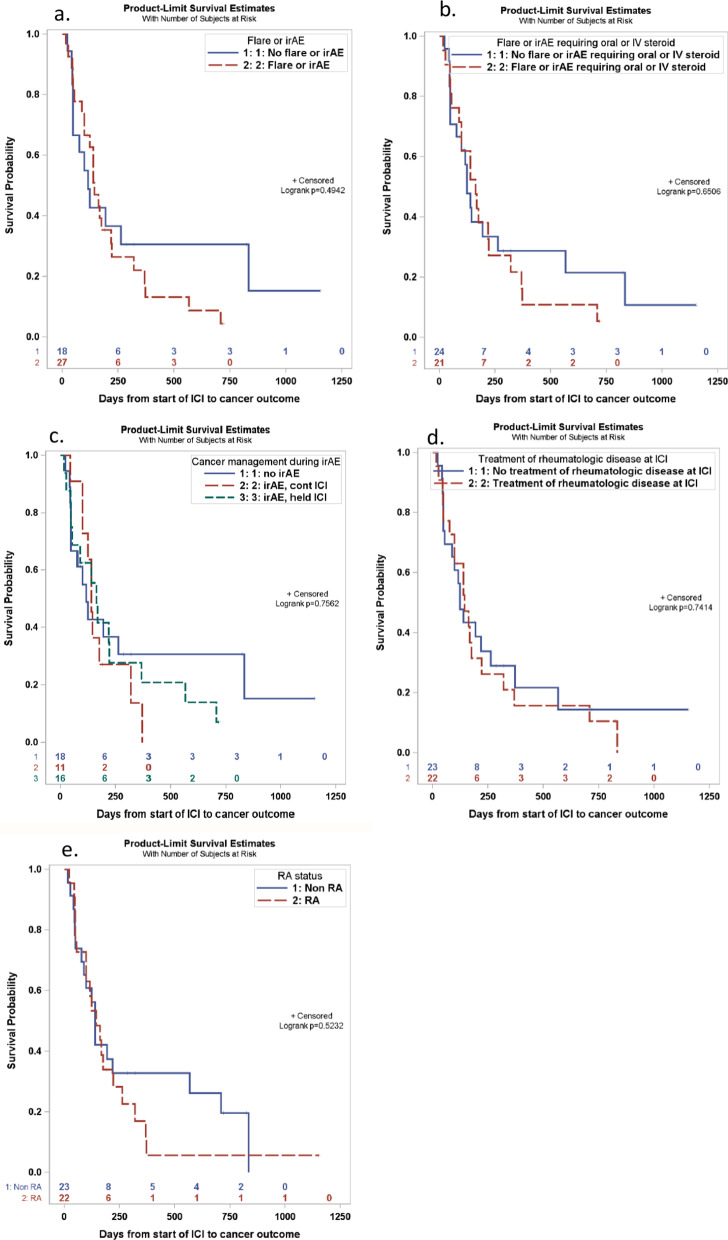
Kaplan–Meier curves comparing time to an unfavorable cancer outcome. Time to an unfavorable cancer outcome (event) based on **a** incidence of a flare or irAE (*n* = 27, 24 events) versus those without (*n* = 18, 13 events), **b** occurrence of a flare of irAE that required oral or intravenous (*n* = 21, 19 events) steroids versus those without an event requiring systemic steroids (*n* = 24, 18 events), **c** ICI being held due to a flare or irAE (*n* = 16, 14 events) versus continued (*n* = 11, 10 events), **d** treatment for the rheumatologic disease during ICI (*n* = 22, 19 events) versus no treatment (*n* = 23, 18 events), and **e** rheumatoid arthritis (*n* = 22, 19 events) versus other rheumatologic disease (*n* = 23, 18 events). None of comparisons showed a statistically significant difference between levels of each stratification factor in time to unfavorable cancer outcomes

Overall, 82% (*n* = 37) of patients in this cohort had an unfavorable cancer outcome (mixed response, progression, death) and there were no complete responders (Fig. 2). Of the 8 patients who did not progress, 4 had lung adenocarcinomas, 1 had small cell lung cancer, 1 had poorly differentiated lung cancer, 1 had breast cancer, and 1 had renal cell carcinoma. Kaplan–Meier curves for time to unfavorable cancer outcome separately by occurrence of a flare or irAE, whether ICI therapy was continued or held after an immune event, administration of systemic steroids for treatment of an immune event, use of anti-rheumatic therapy during ICI treatment, and those with RA versus other rheumatologic diagnoses, are shown in Figs. 3a–e. There were no statistically significant differences in time to an unfavorable cancer outcome between levels of any of these stratification variables. We did not analyze for mortality differences due to the small cohort size and that some patients were lost to follow-up without a reported mortality outcome.

## Discussion

We present an analysis of 45 patients with underlying autoimmune rheumatologic diseases who were treated with ICIs from 2014 to 2019 at UNC. The majority of patients experienced either a disease flare or new onset irAE, which is similar to findings in other cohorts with a variety of autoimmune diseases [12]. The incidence specifically of new-onset irAEs in our cohort (44%) was higher than that seen in a meta-analysis looking at 2392 patients from 23 clinical trials where the incidence of irAEs was 26% in those on anti-PD1 therapy and 14% in those on PD-L1 therapy [19]. In contrast, the incidence of irAEs was higher in those on anti-CTLA-4 therapy in the meta-analysis (54%), but our study only had one patient on this class of medications, so no firm conclusions can be drawn. Raw data were also collected on the 6 patients who received concurrent chemotherapy with ICI treatment. However, the number of patients in this study was too small to meaningfully complete sub-analyses based on concurrent therapy and malignancy type. This would be an area of future direction using a larger cohort with a broader cancer therapeutic regimen including more anti-CTLA-4 and/or concurrent chemotherapy.

Similar to other groups [8, 11, 14, 16, 17], we found a relatively high incidence of underlying disease flares, but not irAEs, in the RA population compared to those with other autoimmune diseases. The incidence of RA flares in these cohorts has ranged from 40 to 75%, showing it is a significant risk that should be addressed with the patient in pre-immunotherapy discussions. Fortunately, RA patients with flares are typically able to continue ICI therapy. For instance, in a prior RA cohort of 22 patients with RA treated with ICIs, 55% had a flare of disease, but only two had to discontinue treatment due to the severity of the reaction [17]. In our cohort, of the 10 patients with an RA flare all events were grade 2 or lower, and only one required the ICI to be held permanently which was confounded by development of malignant pleural effusions and transition to hospice. Seven of the RA patients with a flare responded to prednisone therapy. The higher rate of RA flares in our cohort of patients treated primarily with PD-1 pathway inhibitors may speak to underlying mechanisms in RA pathogenesis. Prior animal studies have shown the development of inflammatory arthritis in PD-1 knockout mice and histopathologic studies showing increased expression of PD-1 and PD-L1 in synovial tissue from RA patients [20–22].

We did not find a statistically significant impact of anti-rheumatic therapy on the incidence of flares, new onset irAEs, or cancer outcomes. There was an interesting trend where the use of anti-rheumatic therapy, primarily hydroxychloroquine in our cohort, was associated with a higher risk of a novel irAE presentation and perhaps a lower risk of a rheumatologic disease flare. This discordance between disease flare and irAE presentations suggest that there may be different mechanisms leading to these disease presentations. At the same time, it is unclear how to extrapolate from this finding given low patient numbers and lack of statistical significance. Since hydroxychloroquine is not known to interfere with T-cell mediated cytotoxicity directly, there is the possibility that these outcomes may have been different if patients were on more aggressive immunosuppression for their rheumatologic disease. While our study focused on examining the rheumatologic population, prior studies have looked at similar questions about the benefit of immunosuppression in patients on ICIs with diverse autoimmune diseases. Regarding incidence of flares, Menzies et al. [14] found a trend towards a higher rate of flares in those not taking concomitant anti-rheumatic therapy; in contrast Leonardi et al. found no association between flares and such therapy, in agreement with the present results. Previous data on the topic of cancer outcomes have been conflicting. Two groups have reported worse progression free survival [11] or response rates [14] in those on anti-rheumatic therapy during ICI treatment, while others have shown no impact of these medications on cancer outcomes [9], in line with our findings.

Malignancy outcomes in this cohort of patients treated with ICIs were poor overall, with only 18% having a favorable response including partial remission or no progression, and no patients having a complete response. This is likely due to the high percentage of patients treated for solid malignancies like NSCLC, where rates of complete remission have been as low as 1.5% [23]. This contrasts with metastatic melanoma where complete remission rates around 16% are reported [24]; our study only included four melanoma patients.

We also looked at cancer outcomes based on the use of systemic steroids for treatment of immune events. The majority of the data to date on the risk of poor cancer outcomes in those on steroids while on ICIs comes from populations without pre-existing rheumatologic disease. In a large meta-analysis, the use of concurrent steroids was associated with increased risk of cancer progression or death [25]. This may reflect the indication for steroid therapy rather than a true medication effect as others have only noted this risk in patients on palliative corticosteroids [26]. We found no association between systemic steroids and poor cancer outcomes suggesting possible confounders in earlier studies. However, we did not stratify by steroid dose, and there is the possibility of a dose effect at which one may see a negative impact. In addition, we did not assess for steroid use under other clinical indications beyond disease flare or irAEs and this may be an area to explore in the future regarding cancer outcome.

While others have shown that the occurrence of a flare or irAE is associated with a better malignancy outcome [3], our study did not show that association, possibly due to limited statistical power. Additionally, by including death due to any cause in our definition of poor malignancy outcomes, we included factors other than progression of disease since some deaths were likely related to infections, co-morbidities, or non-ICI treatment complications.

The limitations of our study, similar to other published cohorts, include limited sample size and lack of a comparator group without rheumatologic autoimmune disease. While the recent publication by van der Kooij et al. [12] included a larger population of patients with autoimmune rheumatologic disease, they focused on grade 3 or higher irAEs, while we included all grades of irAEs and distinguished between disease flare to look at the risks more precisely in this specific patient population. One strategy to potentially capture more patients would be to widen our search criteria to include disease abbreviations. Additionally, since UNC is a tertiary care center, some patients had an offsite rheumatologist which limited historical rheumatologic disease data. For inclusion into this study, all patients had a documented plan of care regarding management of their autoimmune disease detailed in the oncology notes. Due to the retrospective nature of this review and reliance on clinical documentation we were also unable to obtain baseline ECOG scores for all patients, which limited our ability to analyze this important variable that independently correlates with poor outcomes. Another limitation of this study was the lack of detailed documentation on duration and cumulative dose of steroids which prevented a more complete analysis of the effect of steroid use on cancer outcome.

The primary strength of our study is that we completed an in-depth chart review with descriptive analysis. Not only were we able to complete comprehensive mining through a central data repository and utilize search tools to identify cancer patients of interest, but through our descriptive analysis complete a careful review confirming cancer outcomes, rheumatologic flares, and irAE presentations. This is a unique patient population often not included in ICI cancer trials due to concerns for inciting disease flare or heightened risk for irAEs. Our study included a large RA cohort, which allowed for independent analysis on this group. This allowed us to show that RA patients while at higher risk for disease flare, had similar risk for irAEs to general population and no worse outcomes compared to those with other rheumatologic diseases (Fig. 3e).

These results support that patients with stable pre-existing rheumatologic diseases including rheumatoid arthritis should not be excluded from ICI therapy to treat malignancy. To optimize management of disease flare and/or irAEs in cancer patients, it will be helpful to have larger prospective controlled studies to assess true comparative risks in this patient population, establish markers that are predictive for flares and/or irAEs, and determine the best methods of preventing and treating flares and irAEs while preserving cancer treatment efficacy.

## Conclusion

Overall, we showed in our analysis of 45 patients with pre-existing autoimmune rheumatologic diseases treated with ICI therapy that over half of the patients experienced a flare or irAE but the majority of these were not life threating. Continuation of anti-rheumatic therapy during ICI therapy did not prevent disease flares or new onset irAEs, nor did it impact cancer outcome. Further research is needed to determine if there are safe and effective anti-rheumatic therapy options to prevent and treat flares and irAEs in patients with rheumatologic disease on ICI therapy.

## Data Availability

The datasets used and analyzed during the current study are available from the corresponding author on reasonable request.

## Abbreviations

CDW-H: Carolina data warehouse for health
CI: Confidence interval
CTCAE: Common terminology criteria for adverse events
CTLA-4: Cytotoxic T-lymphocyte associated protein 4
DMARD: Disease modifying anti-rheumatic drug
ECOG: Eastern cooperative oncology group
EMERSE: Electronic medical record search engine
I2B2: Informatics for integrating biology and the bedside
ICI: Immune checkpoint inhibitors
irAEs: Immune-related adverse events
NSCLC: Non-small cell lung cancer
OR: Odds ratio
PD-1: Programmed cell death protein 1
PD-L1: Programmed death ligand 1
RA: Rheumatoid arthritis
RD: Risk difference
UNC: University of North Carolina
